# Factors that facilitate multigenerational exchanges in regional locations: a cross‐sectional study in Niigata City, Japan

**DOI:** 10.1186/s13690-021-00563-x

**Published:** 2021-03-25

**Authors:** Kumiko Morita, Minako Kobayashi, Rieko Aoki, Hitomi Nagamine, Harumi Yamamoto, Fumi Ohtake, Mika Hoki, Hiroko Sumita, Kayo Maruyama, Kayoko Mitsuhashi, Akiko Sasaki

**Affiliations:** 1 Tokyo Medical and Dental University (TMDU), 1-5-45 Yushima Bunkyo-ku, 113-8519 Tokyo, Japan; 2 Heisei College of Health Sciences, Gifu, Japan; 3 Wayo Women’s University, Chiba, Japan

**Keywords:** Multigenerational exchanges, Community building, Social capital, Community‐based study

## Abstract

**Background:**

In the process of community building, it is important to create a place for multigenerational exchanges. To promote multigenerational exchanges in regional locations, it is essential to clarify whether such exchanges are related to government infrastructure, regional characteristics, and social capital, and how these exchanges contribute to community building.

**Methods:**

A cross-sectional questionnaire study was conducted with representatives from 455 Chiiki no Cha-no-Ma (literal translation “community living room,” and hereafter “Cha-no-Ma”) in Niigata City, Japan. Responses were received from 405 representatives (response rate: 89.0 %), and 401 agreed to participate (4 declined). The survey details included basic information (e.g., date each location was established, frequency of meetings, number of caretakers and participants, qualifications of the representative), activities reflecting local culture, a social capital scale, the effects of the Cha-no-Ma implemented by the representative (12 items), challenges for management (16 items), and the implementation of multigenerational exchanges.

**Results:**

Most of the age groups that participated in the Cha-no-Ma were elderly, and multigenerational exchanges took place in 125 locations (31.5 %). Items that had a significant connection to the implementation of multigenerational exchanges were “Frequency of meetings” (*p* < 0.001) and “Activities reflecting local culture” (*p* = 0.026). Binomial logistic regression analysis indicated that a high frequency of meetings was associated with the implementation of multigenerational exchanges (Odds ratio = 3.839).

There was a significantly higher ratio of implementation of multigenerational exchanges when the effects were a “connection with the region” (*p* = 0.006) and “conversations with different generations” (*p* = 0.004), and when the challenge was “no support from residents” (*p* = 0.002).

**Conclusions:**

Cha-no-Ma participation is low among young people. The following ideas can be considered in order to increase multigenerational exchanges in regional locations. These exchanges may be promoted by increasing the frequency of meetings with qualified personnel and by adding activities that reflect local culture, such as festivals and making local foods. This community-based study clearly indicates that implementing multigenerational exchanges is an important activity for community building because it is related to connection within the community.

## Background

Japan is facing the prospect of becoming an aging society due to both the extension of life expectancy and the declining birthrate. The average number of family members is predicted to decrease from 2.33 to 2015 to 2.08 by 2040, and the number of solitary households is also increasing [1]. To enable older people to live their lives as they wish in a familiar environment, municipalities and prefectures must establish a community-based integrated care system based on regional autonomy and independence [2]. The need for a community-based integrated care system is urgent because the number of family members available to support elder care is increasingly limited. In the process of community building, it is important to create places where people may interact. Over the past several decades, social isolation and loneliness among older adults have posed an increasingly urgent challenge because of the rapidly aging population in Japan. To remedy the situation, many communities have introduced multigenerational programs. Accordingly, attention has been focused on the Chiiki no Cha-no-Ma (hereafter “Cha-no-Ma”), which have been implemented in Niigata City in Niigata Prefecture [3].

Niigata City has a population of approximately 800,000, and it is almost 2 h by bullet train from Tokyo. The proportion of persons aged over 65 years was 29.2 % in 2019. Niigata is famous as a rice-producing area of Japan, but there is a shortage of farmers as many young people have left the region to live in cities. In 1964, the area experienced a magnitude 7.5 earthquake, which caused significant damage. However, inhabitants cooperated and rebuilt the city. Community ties and civil society activities are not only decisive in fostering community resilience against disaster, but also for coordinating relief, rebuilding, and by extension, adapting to perpetual change [4].

“Chiiki no Cha-no-Ma” can be translated literally as “community living room.” It is not a religious or political organization, but rather a place where older people and mothers with children in the neighborhood can easily visit and spend a pleasant time with people of different age groups. The phrase “third place” has been defined as “public places that host the regular, voluntary, informal, and happily anticipated gatherings of individuals beyond the realms of home and work” [5]. Cha-no-Ma is one such third place. In 1997, Cha-no-Ma began with monthly regional exchanges in local community halls. Cha-no-Ma received attention for being locations that, rather than offering special programs, permitted local residents to visit and spend as much time there as they wished. Cha-no-Ma have developed as a form of citizen-led support using meeting places and vacant homes. With cooperation from social welfare councils and the Welfare Division of Niigata City, at least 500 locations were operating in Niigata as of 2018, with the involvement of specialists such as public health nurses, hospital nurses, and occupational therapists. The 2014 revision of the “Guidelines for health activities of public health nurses in the community” [6] included the promotion of self-help and mutual support using social capital (e.g., community-based trust, social standards, networks, and society-related capital), and identified Cha-no-Ma as a base for regional activities by public health nurses.

Furthermore, multigenerational exchange effects are expected as different generations gather at Cha-no-Ma. Multigenerational exchange denotes that members of different generations can be present, feel welcome, and engage in activities [7]. Many of the effects of intergenerational exchanges have been revealed in prior studies that focused on older persons and children [8–13]. Moreover, Cha-no-Ma are expected to foster community regeneration based on the connections created between citizens through intergenerational exchange; studies have assessed their positive effects on increasing social capital (hereafter “SC”) [14–17]. SC and health is a multidisciplinary topic, with studies often drawing from theories and concepts in the social, political, and behavioral sciences [18]. Putnam [19] defined SC as the features of social organizations, such as networks, norms, and social trust, that facilitate coordination and cooperation for mutual benefit. Carpiano [20] suggested that SC consists of four main forms: (1) social support, (2) social leverage, (3) informal social control, and (4) neighborhood organization and participation.

Cha-no-Ma are spaces where people from different generations can socialize and interact rather than be isolated, which ideally produces spontaneous multigenerational exchanges [21]. However, the current situation is that most participants are older people; an important issue, therefore, is how to promote participation by members of younger generations so that multigenerational exchanges can occur in Cha-no-Ma. Implementing multigenerational exchanges in after-school care is expected to promote “learning experiences and knowledge from older persons” and “nurturing respect for older persons.” However, talent acquisition (individuals who can act as facilitators of multigenerational exchanges) and location are issues that must be addressed [22]. Nevertheless, while after-school care is a government-sponsored activity, Cha-no-Ma is run by community members. Can a government infrastructure influence the implementation of multigenerational exchanges?

The community-based integrated care system seamlessly provides healthcare, long-term care, preventive care, housing, and livelihood support services so that older people can live independently in their communities. However, this is not a nationwide approach, and it is carried out independently by each region [2]. Depending on the region, there are areas where residents have strong ties with each other, and there are areas where even neighbors rarely meet. Can regional characteristics influence multigenerational exchange? It has been proposed that SC is strengthened through the activation of civic activity, and that civic activity is promoted if SC is rich [23]. While the former has been confirmed in previous research, the positive influence of rich SC on multigenerational exchange has not been confirmed. Cha-no-Ma is modeled in many areas and is highly useful in clarifying how multigenerational exchanges that deepen community ties are carried out therein.

Therefore, in this study, we clarified whether government infrastructure, regional characteristics, and SC are related to the implementation of multigenerational exchange in Cha-no-Ma. Further, we clarified their relationship with the effects and challenges of Cha-no-Ma, investigating how the implementation of multigenerational exchange can contribute to community building.

## Methods

### Participants

The study covered 455 Cha-no-Ma locations that were active as of July 2019, of which 405 responded (response rate: 89.0 %) and 401 agreed to participate (4 declined). The study period was June to October 2019.

### Design

This was a cross-sectional study based on a self-report questionnaire. The distribution of the questionnaires was conducted by Niigata City, which organizes the Cha-no-Ma, and the questionnaires were returned directly to the researchers after completion.

### Outcome measures

**Fig. 1:**
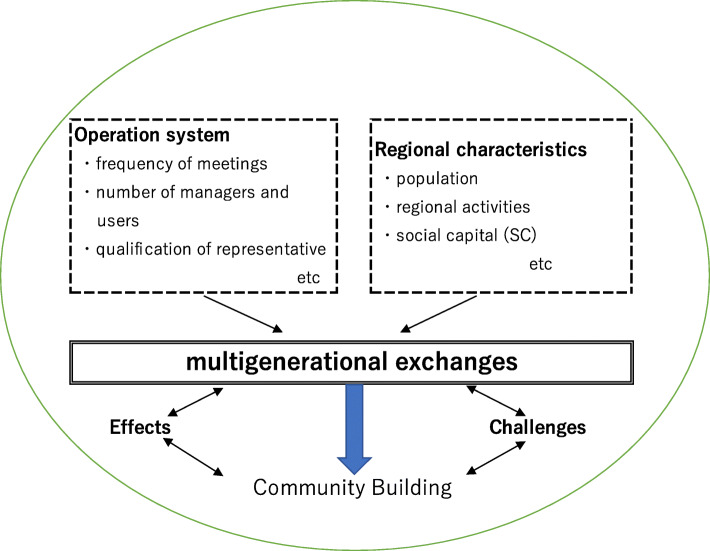
Brief conceptual overview

The survey included basic information (e.g., date facility was established, frequency of meetings, number of caretakers and participants, qualifications of the representatives), activities reflecting local culture, a social capital scale (hereafter “SC Scale”), the effects of the Cha-no-Ma being implemented by the representatives (12 items), challenges for management (16 items), and the implementation of multigenerational exchanges. A conceptual overview is shown in Fig. 1.

The SC Scale developed by Kawaharada et al. [24] comprises 20 items, and measures the outcome of community health activities related to the development of SC. Many conventional SC scales measure the status of interactions among individual residents in their areas. In contrast, the scale developed by Kawaharada et al. includes items that measure the situation of the entire area and the relationship with the public health nurse or nutritionist. The representative of the Cha-no-Ma has a close relationship with the area and can likely evaluate the SC of the entire area, hence the choice to use this scale. The internal validity was confirmed, with a Cronbach’s α coefficient of 0.92. Moreover, this scale permitted analysis of each factor. In this study, three factors were used to examine the residential region from the perspective of the representative, namely, Factor 1: Trust and support of local people (7 items), Factor 3: Affinity with city professions (3 items), and Factor 5: Association with the neighborhood (3 items). Answers for items were provided on a 5-point Likert scale, with response options of “Strongly agree” (5 points), “Agree,” “Neither agree nor disagree,” “Mostly disagree, and “Disagree” (1 point). A higher score indicates better SC conditions. The items addressing the effects of, for example, regional connections or improvement of physical and cognitive functions and management challenges, such as a deficit in profits of transportation to Cha-no-Ma, were created with reference to prior studies [22, 25], with multiple selections allowed.

### Operational definitions

Multigenerational exchange: In this type of exchange, it is assumed that two or more generations interact with one another.

Representatives: They are managers of the Cha-no-Ma and support on-site programming. They are residents rather than city employees, but operate in collaboration with Niigata City through an agreement. Some have specialist qualifications (e.g., nurse), but these qualifications are not a requirement for the role.

Caretakers: They are residents who support the operation of the Cha-no-Ma along with representatives. In many cases, they participate as paid or unpaid volunteers.

Participants: They are people from the community, regardless of age, who attend the Cha-no-Ma programming.

Specialists: Depending on the Cha-no-Ma, specialists such as public health nurses and occupational therapists visit regularly to check participants’ health. The specialist is a different from the representative.

### Data analysis

Statistical analysis was performed using IBM SPSS Statistics version 25 for Windows. A *p*-value of < 0.05 was considered statistically significant. To compare differences in characteristics between two groups, we employed Fisher’s exact test and the Mann–Whitney U test. Logistic regression analysis was carried out with the implementation of multigenerational exchanges as the dependent variable and the related factors as independent variables.

### Ethics approval and consent to participate

All participants were informed about this study in writing before it commenced. Consent for participation was assumed based on the return of questionnaires. We received approval from the ethics review board of Tokyo Medical and Dental University (Approval number: M2018-318; approved on April 19, 2019).

## Results

### Operational status of cha‐no‐ma

**Fig. 2:**
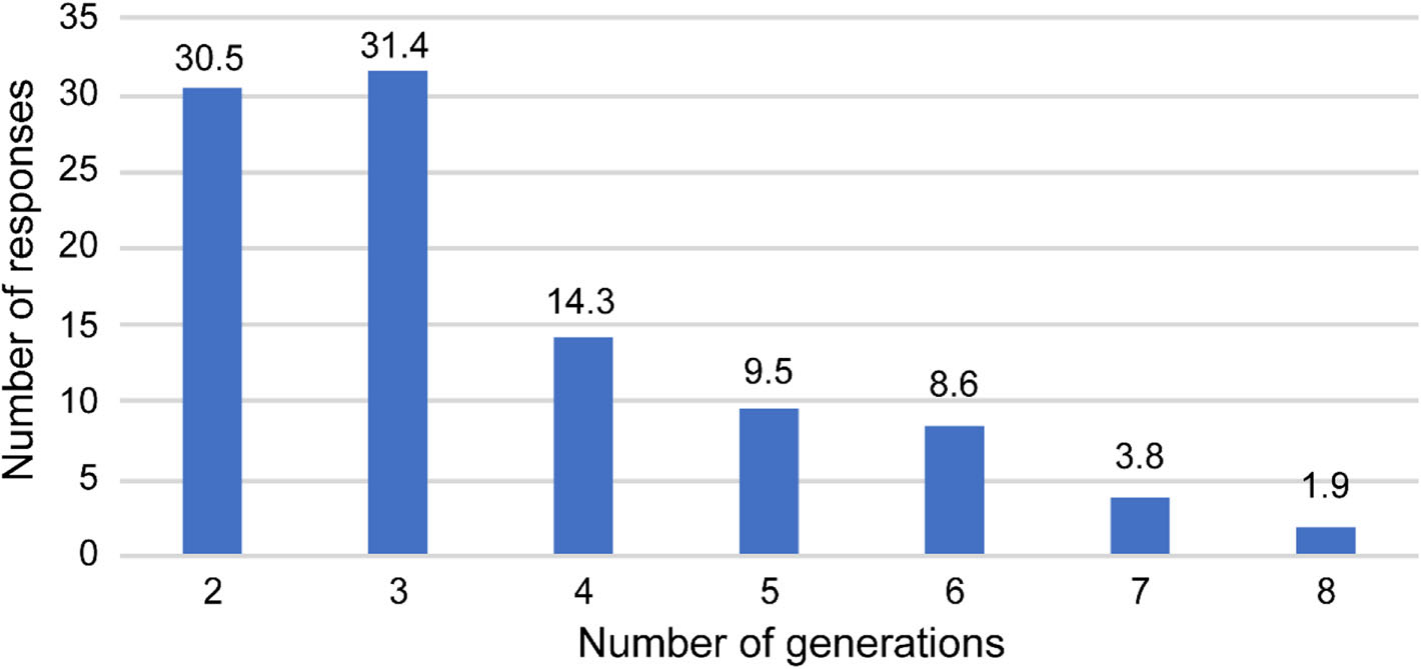
Number of generations interacting

Table 1 shows the operational status of Cha-no-Ma. More than half of the Cha-no-Ma (236; 66.1 %) were established in 2010 or thereafter. Approximately 90 % of the participants were 75 years of age or older, or between 65 and 74. Multigenerational exchanges were said to take place in 125 locations (31.5 %). Of the 125 cases in which multigenerational exchanges were conducted, 32 (30.5 %) occurred with two generations and 73 (69.5 %) with 3 or more generations (excluding no answer) (Fig. 2).

**Table 1:** Operational status of Cha-no-Ma

		n	%
Frequency of meetings	Once or more per week	57	14.2
	Once/twice per month	344	85.8
Year established	Prior to 1999	26	7.3
	2000–2009	95	26.6
	2010–2019	236	66.1
Age groups engaged at Cha-no-Ma: multiple answers per establishment	75 or above	357	90.6
	65–74	353	89.6
	40–64	145	36.8
	18–39	36	9.1
	Senior high school student	6	1.5
	Junior high school student	15	3.8
	Elementary school student	59	15.0
	Pre-school-age child	28	7.1
Multigenerational exchanges	Yes	125	31.5
Average number of participants per day		18.0 ± 8.3	
Average number of caretakers		4.1 ± 3.1	
Average age of representative		72.3 ± 7.1	

### Factors related to the implementation of multigenerational exchanges

Table 2 shows the items significantly associated with implementation of multigenerational exchanges. The ratio of multigenerational exchanges was significantly higher (*p* < 0.001) in groups that met more frequently (once or more per week). Of the 135 specific descriptions of activities reflecting local culture, the main items were related to food (49 items), such as “making local foods,” followed by items related to “festivals” (36 items).

**Table 2:** Items significantly associated with implementation of multigenerational exchanges

			Multigenerational exchanges present		
Item		Number of responses: excluding no answer	n	%	*p*-value^a^
Frequency of meetings	High (once or more per week)	56	32	57.1	< 0.001
	Low (once/twice per month)	341	93	27.3	
Specialist qualification of representative (e.g., nurse)	Yes	78	32	41.0	0.026
	No	279	77	27.6	
Activities reflecting local culture (e.g., making local foods, festivals, seasonal events)	Yes	147	60	(40.8)	0.001
	No	217	53	(24.4)	

^a^ Fisher’s exact test

Table 3 shows the items that differed significantly by Mann–Whitney U Test. The median number of caretakers was 4.5 in Cha-no-Ma in which multigenerational exchanges were implemented, and 3.5 otherwise (*p* = 0.003). The effect size was 0.22 to 0.48.

**Table 3:** Items that differed significantly according to whether multigenerational exchanges were implemented

	ME	Average rank	Median	*p*-value^a^	*d*^b^
Number of caretakers	yes	221.44	4.50	0.003	0.26
	no	185.10	3.00		
SC score (Factor I)	yes	209.77	27.00	0.003	0.31
	no	174.14	26.00		
SC score (Factor V)	yes	209.49	12.00	0.032	0.22
	no	183.83	12.00		
Total SC score	yes	202.12	49.00	0.015	0.48
	no	173.32	47.00		

^a^ Mann–Whitney U test

^b^ Effect size: This was calculated using the method developed by Cohen (1988)

Abbreviations: *ME* multigenerational exchange; *SC* social capital

Among the relevant factors clarified in Tables 2 and 3, a binomial logistic regression analysis was conducted to confirm the extent and the influence of multigenerational exchanges (Table 4). With consideration of collinearity, only the total SC scale score was used for the items that were significant in the univariate analysis. Among the other items, there were no correlations of *r* > 0.2. A high frequency of meetings was associated with the implementation of multigenerational exchanges (Odds ratio, OR = 3.839).

**Table 4:** Factors related to multigenerational exchanges (multivariate analysis)

	B	SE	Wald	*p*-value	OR	OR 95% confidence interval	
						Minimum	Maximum
High frequency of meetings	1.345	0.337	15.982	<0.001	3.839	1.985	7.425
No. of caretakers	−0.074	0.047	2.513	0.113	0.929	0.848	1.018
Specialist qualification^a^ of representative	0.785	0.297	6.998	0.008	2.193	1.226	3.925
SC scale total	−0.030	0.019	2.669	0.102	0.970	0.936	1.006
Activities reflecting local culture	0.585	0.267	4.806	0.028	1.794	1.064	3.027
Constant	0.515	0.993	0.269	0.604	1.674		

^a^ Specialist qualifications included nurses, doctors, childcare workers, nutritionists, counselors, teachers, etc.

Binomial logistic regression analysis: Forced entry method; Model χ^2^, *p* < 0.001, Hosmer–Lemeshow test, *p* = 0.572.

Abbreviation: *OR* odds ratio; *SE* standard error

### Relationship between the effects of cha‐no‐ma and the implementation of multigenerational exchanges

There were associations between the effects of Cha-no-Ma and implementation of multigenerational exchanges for 2 of 12 items (Table 5). Implementation of multigenerational exchanges was significantly more likely given a “connection with the region” (*p* = 0.006) and “conversations with different generations” (*p* = 0.004). Next, the analysis was conducted only in places where multigenerational exchanges were carried out. When comparing the two-generation exchange with the three-generation or more (3+) exchange, the 3 + generation exchange showed significantly greater “improvement of cognitive function,” “interest in health,” “increased smiling,” and “conversations with different generations” (*p* < 0.05; Table 5).

**Table 5:** Relationship between the effects of Cha-no-Ma participation and multigenerational exchanges

		All data (excluding no answer)				Only those implementing multigenerational exchange (excluding no answer)			
			Multigenerational exchanges found				3+ generation exchanges found		
Effect of Cha-no-Ma		Number of responses	n	%	*p*-value^a^	Number of responses	n	%	*p*-value^a^
Increased smiling	Effect	314	96	30.6	0.500	79	59	74.7	0.045
	No effect	80	28	35.0		26	14	53.8	
Interest in health	Effect	275	88	32.0	0.813	74	58	78.4	0.005
	No effect	118	36	30.3		31	15	48.4	
Connection with the region	Effect	264	95	36.0	0.006	80	55	68.8	0.809
	No effect	130	29	22.3		25	18	72.0	
Prevention of isolation	Effect	252	80	31.7	0.910	67	50	74.6	0.185
	No effect	142	44	31.0		38	23	60.5	
Conversations with same generations	Effect	232	77	33.2	0.442	67	47	70.1	1.000
	No effect	162	47	29.0		38	26	68.4	
Watch over	Effect	194	70	36.1	0.440	59	45	76.3	0.134
	No effect	200	54	27.0		46	28	60.9	
Improved physical function	Effect	133	46	34.6	0.360	37	29	78.4	0.185
	No effect	261	78	29.9		68	44	69.7	
Attend alone	Effect	132	47	35.6	0.250	37	26	70.3	1.000
	No effect	262	77	29.4		68	47	69.1	
Improved cognitive function	Effect	99	27	27.3	0.319	21	19	90.5	0.019
	No effect	295	97	32.9		84	54	64.3	
Conversations with different generations	Effect	96	42	43.8	0.004	39	33	84.6	0.015
	No effect	298	82	27.5		66	40	60.6	
Meal support	Effect	57	18	31.6	1.000	14	9	64.3	0.756
	No effect	337	106	31.5		91	64	70.3	
Read newspapers or magazines	Effect	9	3	33.3	1.000	3	3	100.0	0.551
	No effect	385	121	31.4		102	70	68.6	

^a^ Fisher’s exact test

### Relationship between challenges of cha‐no‐ma and the implementation of multigenerational exchanges

There were associations between the implementation of multigenerational exchanges and the challenges of implementation for 4 of 16 items (Table 6). Implementation of multigenerational exchanges was significantly more likely when facing the challenge of “no support from residents” (*p* = 0.002).

**Table 6:** Relationship between the challenges of Cha-no-Ma and implementation of multigenerational exchanges

			Multigenerational exchanges found		
Challenge		Number of responses: excluding no answer	n	%	*p*-value^a^
Fixed participants	Yes	227	67	29.5	0.372
	No	158	54	34.2	
Fostering successors	Yes	198	68	34.3	0.228
	No	187	53	28.3	
Participants do not increase	Yes	196	58	29.6	0.444
	No	189	63	33.3	
Expected age group does not come	Yes	152	55	36.2	0.116
	No	233	66	28.3	
Securing caretakers	Yes	136	47	34.6	0.359
	No	249	74	29.7	
Difficult to enroll new participants	Yes	99	37	37.4	0.167
	No	286	84	29.4	
Lack of operational know-how	Yes	46	17	37.0	0.401
	No	339	104	30.7	
No challenges	Yes	33	10	30.3	1.000
	No	352	111	31.5	
Forming networks with other managers	Yes	25	13	52.0	0.027
	No	360	108	30.0	
Participant relationships	Yes	25	10	40.0	0.375
	No	360	111	30.8	
Not well known	Yes	24	8	33.3	0.823
	No	361	113	31.3	
Poor transportation access	Yes	22	12	54.5	0.030
	No	363	109	30.0	
No support from local residents	Yes	20	13	65.0	0.002
	No	365	108	29.6	
Deficit in profits	Yes	19	11	57.9	0.020
	No	366	110	30.1	
Difficult to secure an exchange place	Yes	10	3	30.0	1.000
	No	375	118	31.5	
The effect of the activity is small or unclear	Yes	8	3	37.5	0.710
	No	377	118	31.3	

^a^ Fisher’s exact test

## Discussion

### Factors related to the implementation of multigenerational exchanges

As shown in Table 4, higher frequency of holding meetings was the primary variable associated with implementation of multigenerational exchanges. It is likely easier for children and working people to arrange their schedules if meetings are held frequently. Morita et al. [22, 26] also reported that the implementation and continuation of exchanges between generations are affected by schedule adjustments. Furthermore, the amount of funding from the government in Niigata City depends on the frequency of meetings held. While locations that hold meetings once or more per week receive up to 20,000 yen (~ 190 US$/160 euros) per month as well as an initial payment of 200,000 yen (~ 1,900 US$/1,600 euros), those locations that hold meetings once or twice per month receive a payment of only up to 2,500 yen (~ 25 US$/20 euros) per month [27]. Pain et al. [28] suggested that “More direct funding is needed for intergenerational programs” and “Financial support and social policy support are critical for the long-term sustainability of intergenerational programs” [29, 30]. It was assumed that the budget would have the same connection in the current study; groups that meet once or more per week and receive a larger subsidy are likely to have better environments for implementing multigenerational exchanges.

Even if there is a desire to implement multigenerational exchanges, this cannot be enacted if there is a lack of applicable knowledge. In this study, many of the representatives with specialist qualifications were nurses, doctors, childcare workers, nutritionists, or counselors. The success of intergenerational programs is determined by the presence of a skilled healthcare professional and a qualified recreation therapist [31]. The most significant subcomponents regarding a facilitator’s skills include being knowledgeable about different generations (e.g., children, youth, and older persons) and having attended formal training on intergenerational program management [32]. Considerable current research in the field examines the importance of knowledge and behaviors of persons who implement the multigenerational programming [33, 34]. As shown in these reports, a facilitator should be present when multigenerational exchanges are implemented. However, because of the current lack of personnel [22, 35], representatives’ specialist qualifications affect the implementation of multigenerational exchanges. For example, if a representative has a nursing qualification, it will be useful for considering health.

Several activities reflected local culture, such as cooking together using local foods. Exchanges that incorporated the theme of food were well received by all generations. Indeed, a prior report indicated that “The food-based activities worked well in bringing the groups together” [36]. Furthermore, multigenerational exchanges occurred through festivals based in the region. Festivals are tools of community gathering, intergenerational communication, and transmission of knowledge to the younger generations [37]. Food and festivals can be used to plan exchanges between generations.

It was expected that those Cha-no-Ma with higher SC would have more multigenerational exchanges, but no such significance was found by multivariate analysis. The effect size of SC was 0.48 in Table 3, and the value of the effect size in a two-group test of mean differences is estimated at 0.50 for medium effects [38]. Hence, increasing the number of respondents may be significant in multivariate analysis. Since SC is becoming diluted in society, the operational system may have greater impact than SC on the implementation of multigenerational exchanges. That is, it is possible to carry out multigenerational exchanges by establishing an operational system even in areas where SC is not established.

### Effects of multigenerational exchanges

In this study, those who implemented multigenerational exchanges reported an effect of “connection with the region.” The effect was more pronounced in multigenerational exchanges than in two-generation exchanges. Multigenerational exchanges provide insight into intergenerational diversity and may foster problem solving for individuals, families, and communities. Exchanges between generations had the same effects as reported in prior studies, namely a decrease in the risk of social isolation [7, 39] and an expansion of social networks [40–42]. Furthermore, according to the 2019 Annual Report on the Aging Society [43], most persons aged 60 or over stated that “support to and from neighbors” was necessary to continue living happily in the region where they currently reside (55.9 %). Considering the different types of cohabitation, there was a higher ratio of two- or three-generation households living with their parents than single-person households or married couples, which shows not only that older persons require support within their region, but also that families with children and even grandchildren feel that such support is necessary. Community building requires the creation of connections between residents and with the region [44], and the multigenerational exchanges conducted at Cha-no-Ma can be considered useful in this regard.

The implementation of multigenerational exchanges also increases conversations among different generations, as revealed in prior studies [41]. In terms of exchange methods, interactive programs increase the conversation frequency [8]. Conversations among different generations increase the knowledge level of each party more than interactions within the same generation [45]. Conversation may prevent cognitive deterioration among older persons [34, 46], improve attitudes toward older people [11, 12, 31, 47–51], and lead to the transmission of knowledge to children [22, 45]. Furthermore, communication is an important factor in SC [17]; communication at Cha-no-Ma connects people so that conversations arise naturally in locations other than Cha-no-Ma. This may promote residents’ safety by preventing crime through observing surroundings and communicating with each other.

### Challenges posed by the implementation of multigenerational exchanges

While the implementation of multigenerational exchanges was found to have positive outcomes, it was also clarified that such exchanges posed challenges. In the 2018 survey of “Generations United,” challenges were reported regarding demonstrating the impact of intergenerational programs (63 %), funding intergenerational programming (60 %), and ensuring accessibility of spaces for all participants (48 %) [52]. Schemes such as enhancing programs for the provision of meals and the planning of festivals are required to implement multigenerational exchanges, and they involve a large budget. Therefore, it is important to secure a suitable budget [28–30]. Regarding the problem of transport access, it may be necessary to appeal to the city to introduce transportation services. A solution to the challenge of “no support from residents” may be found by proactively sharing information. The same is true for “forming networks with other managers,” which was also cited as a challenge. According to Ayala et al. [53], there is a need to network, collaborate, and/or partner with other organizations. In Niigata City, a “Cha-no-Ma School” is held every year to train the personnel who manage regional Cha-no-Ma [54]. Additionally, examples of activities are compiled [55] and published on the city website. It is expected that the network of representatives will expand in the future.

### Study limitations

This survey was limited to Cha-no-Ma implemented in Niigata City; therefore, the results may not apply to other regions or countries. It is necessary to study the effects and challenges of implementing multigenerational exchanges in other regions, considering the characteristics, environment, and culture of each region. While this was a cross-sectional, quantitative study, it may be possible to gain more detailed information by conducting a qualitative study.

## Conclusions

Multigenerational exchange in a regional location could be promoted by increasing the frequency of meetings with personnel holding qualifications in areas such as healthcare, childcare, or education. Further, it would be effective to use activities that reflect local culture, such as making local foods and organizing festivals. It is necessary to establish programs that interest participants. Future challenges include cooperating with the government to secure financial resources and transportation access. Actively disseminating case studies will help residents to understand and build networks collaboratively with other operators. It is clear from this community-based study that the implementation of multigenerational exchanges is an important activity for community building because it is related to an increase in communication among generations and connection within the community.

## Data Availability

The data sets used and/or analyzed during the current study can be made available by the corresponding author on reasonable request.

## Abbreviations

SC: Social capital
ME: Multigenerational exchange
OR: Odds ratio
SE: Standard error
